# Frontal Dysfunctions of Impulse Control – A Systematic Review in Borderline Personality Disorder and Attention-Deficit/Hyperactivity Disorder

**DOI:** 10.3389/fnhum.2014.00698

**Published:** 2014-09-03

**Authors:** Alexandra Sebastian, Patrick Jung, Annegret Krause-Utz, Klaus Lieb, Christian Schmahl, Oliver Tüscher

**Affiliations:** ^1^Emotion Regulation and Impulse Control Group, Focus Program Translational Neuroscience, Department of Psychiatry and Psychotherapy, Johannes Gutenberg-University, Mainz, Germany; ^2^Department of Psychosomatic Medicine and Psychotherapy, Central Institute of Mental Health Mannheim, Medical Faculty Mannheim, Heidelberg University, Mannheim, Germany; ^3^Department of Neurology, Albert-Ludwigs-University Medical Center, Freiburg, Germany

**Keywords:** impulsivity, response inhibition, borderline personality disorder, attention-deficit/hyperactivity disorder, fMRI

## Abstract

Disorders such as borderline personality disorder (BPD) or attention-deficit/hyperactivity disorder (ADHD) are characterized by impulsive behaviors. Impulsivity as used in clinical terms is very broadly defined and entails different categories including personality traits as well as different cognitive functions such as emotion regulation or interference resolution and impulse control. Impulse control as an executive function, however, is neither cognitively nor neurobehaviorally a unitary function. Recent findings from behavioral and cognitive neuroscience studies suggest related but dissociable components of impulse control along functional domains like selective attention, response selection, motivational control, and behavioral inhibition. In addition, behavioral and neural dissociations are seen for proactive vs. reactive inhibitory motor control. The prefrontal cortex with its sub-regions is the central structure in executing these impulse control functions. Based on these concepts of impulse control, neurobehavioral findings of studies in BPD and ADHD were reviewed and systematically compared. Overall, patients with BPD exhibited prefrontal dysfunctions across impulse control components rather in orbitofrontal, dorsomedial, and dorsolateral prefrontal regions, whereas patients with ADHD displayed disturbed activity mainly in ventrolateral and medial prefrontal regions. Prefrontal dysfunctions, however, varied depending on the impulse control component and from disorder to disorder. This suggests a dissociation of impulse control related frontal dysfunctions in BPD and ADHD, although only few studies are hitherto available to assess frontal dysfunctions along different impulse control components in direct comparison of these disorders. Yet, these findings might serve as a hypothesis for the future systematic assessment of impulse control components to understand differences and commonalities of prefrontal cortex dysfunction in impulsive disorders.

## Impulsivity as a Diagnostic Criterion

Impulsivity is regarded as a clinical, diagnostic, and pathophysiological hallmark of several neuropsychiatric disorders such as borderline personality disorder (BPD), attention-deficit/hyperactivity syndrome (ADHD), obsessive–compulsive disorder, trichotillomania, pathologic gambling, and chronic substance abuse (Chamberlain and Sahakian, 2007; Aron, 2011). A complete review of frontal dysfunctions associated with impulsivity across the whole range of psychiatric disorders is beyond the scope of this review. We will therefore focus on frontal dysfunctions in BPD and adult ADHD and their relation to different components of impulse control.

In BPD, impulsivity is a central symptom and key component of neurobehavioral models of the disease (Lieb et al., 2004). According to the Diagnostic and Statistical Manual of Mental Disorders (DSM-5; APA, 2013) impulsivity in at least two potentially self-damaging areas such as excessive spending, sex, substance use, binge eating, reckless driving, or physically self-damaging acts is required to fulfill the diagnostic criterion. In the recently released DSM-5 (APA, 2013), it has been proposed that impulsive, self-harming behavior may occur (mainly) under emotional distress. This is an important advancement of diagnostic criteria as impulsive behavior in BPD appears to be substantially modulated by negative, especially by BPD-salient emotions (Sebastian et al., 2013a).

In ADHD, one of the main diagnostic symptoms besides inattention and hyperactivity is impulsivity (APA, 2013). Impulsive behaviors may consist of blurting out answers before questions have been completed, having difficulties awaiting a turn, or interrupting or intruding on others (APA, 2013). As most of the symptoms listed in DSM-5 are rather observed in childhood ADHD other impulsive symptoms have been suggested for adult ADHD such as impatience (e.g., while driving) or impulsive buying. Other major manifestations of adult ADHD are thought to be poor occupational performance, abrupt initiation or termination of relationships (e.g., multiple marriages, separations, divorces), and excessive involvement in pleasurable activities without recognizing risks of painful consequences etc. (Wender et al., 2001). Similarly, additional impulsive symptoms have been added in the DSM-5 (APA, 2013), such as leaving the place in the office in a situation in which one is expected to remain seated.

As one may deduce from the multiple impulsive symptoms listed above, there is no commonly accepted unitary definition of impulsivity in the clinical domain even though impulsivity is considered to be a diagnostic criterion for several psychiatric disorders (Moeller et al., 2001). The assessment of underlying neural dysfunctions is further complicated by multifaceted nature of impulse control (Dalley et al., 2011; Sebastian et al., 2013b; Stahl et al., 2014) that will therefore be addressed in the next section.

## The Multifaceted Nature of Impulse Control

### Components of impulse control

Impulse control as an executive function is neither cognitively nor neurobehaviorally a unitary function (Sebastian et al., 2013b; Stahl et al., 2014). Recent findings from behavioral and cognitive neuroscience studies suggest related but dissociable components of impulse control along functional domains such as selective attention, cognitive control, response selection, motivational control, and behavioral inhibition (Friedman and Miyake, 2004; Nee et al., 2007; Cyders and Coskunpinar, 2011; Dalley et al., 2011; Stahl et al., 2014). Using a structural-equation modeling approach, Stahl et al. (2014) recently demonstrated that at least six separable but related components of impulse control exist: the control of stimulus interference, proactive interference, response interference, and behavioral inhibition as well as decisional and motivational impulsivity. It should be noted that varying conceptualizations and definitions of impulse control components have been suggested [for overview see Dalley et al. (2011), Bari and Robbins (2013), and Stahl et al. (2014)].

(1) Stimulus interference may be defined as the ability to suppress or resolve interference due to resource or stimulus competition related to information in the external environment that is irrelevant to the task at hand (Friedman and Miyake, 2004; Nee et al., 2007). Thus stimulus interference may be considered as impulse control at an attentional level. In stimulus interference tasks such as the Stroop paradigm, participants assess whether a probe stimulus matches a target stimulus (Stahl et al., 2014).
(2) Proactive inhibition consists of the suppression of information that was previously relevant to the task but has since become irrelevant (Nigg, 2000; Friedman and Miyake, 2004). As this impulse control component requires control of information in working memory it may be assigned to impulse control at a cognitive level. Proactive inhibition may be assessed using the recent probes task or the directed forgetting task (Stahl et al., 2014).
(3) Impulse control may occur at different behavioral levels. Response interference may result from the activation of irrelevant response tendency (Stahl et al., 2014) and response priming as well as task-switching paradigms have been shown to almost exclusively reflect response-related interference (Klauer et al., 2005).
(4) Whereas response interference rather involves competition between two task-relevant responses and, thus, interference is present at an earlier response-selection stage, behavioral inhibition focuses on withholding or cancelation of an already selected or initiated response, and thus, late control processes (Sebastian et al., 2013b; Stahl et al., 2014). Stop-signal- and go/no-go tasks belong to the most prominent behavioral inhibition tasks (Aron, 2011; Swick et al., 2011; Sebastian et al., 2013b).
(5) Impulse control may also be necessary at a decisional level. This component is represented by information sampling, which relates to a decision-making style and assesses the amount of information sampled before a decision is reached (Kagan, 1966; Bechara, 2005; Stahl et al., 2014). Impulse control in the sense of a lack of reflection can be assessed by measuring participants’ response criterion, which can be rather liberal or conservative. High impulsivity is assumed to be associated with a relatively liberal criterion; an impulsive decision is made when a person samples only a small amount of information (Bechara, 2005; Stahl et al., 2014).
(6) A motivational component of impulse control consists of the temptation of short-term reward, thereby interrupting long-term goals to the degree that delayed rewards are discounted. Delay of gratification may best be assessed by delay discounting paradigms (Dalley et al., 2011; Mischel et al., 2011; Stahl et al., 2014).

In addition to the multifaceted components of impulse control, it becomes increasingly evident that some of the disparities result from the variety of methods that are being used to assess impulse control [for example and discussion see Cyders and Coskunpinar (2011)]. Besides experimental paradigms as those listed above impulsivity or, more precisely, personality traits of impulsivity can be assessed using self-report measures. However, if at all present correlations between impulsivity traits as assessed using self-report scales and state impulsivity as assessed using experimental paradigms are relatively small (Reynolds et al., 2006; Jacob et al., 2010; Cyders and Coskunpinar, 2011; Stahl et al., 2014).

### Proactive and reactive inhibitory control

Besides the division into its various components, impulse control can be distinguished by different control strategies, according to the dual mechanisms of control (DMC) model (Braver et al., 2007; Braver, 2012). Attention, perception, thoughts, and actions are controlled proactively or reactively, depending on the usage of prior knowledge and cues that navigate the expectation level of upcoming events. On a behavioral level, highly predictive cues and sustained active maintenance of task goals permit the proactive execution or inhibition of actions whereas unexpected salient stimuli implement reactive behavioral control. In contrast to reactive control, proactive control should (i) enable facilitated, more selective, and more accurate actions, (ii) protect from distracting, goal-irrelevant stimuli, and (iii) be favorable to protect the individual from actions that are potentially harmful to the self and/or others. However, proactive control is limited by (i) its reliance upon the presence of highly predictive contextual cues, (ii) its high sustained metabolic demand to actively maintain goal-relevant information, and (iii) its limited capacity since only a small number of goals can be actively maintained (Cowan, 2001; Braver et al., 2007; Greenhouse et al., 2012). The latter feature of proactive control suggests a close linkage to working memory capacity and fluid intelligence (Fry and Hale, 2000; Kane et al., 2005; Oberauer et al., 2005). Indeed, there is evidence for an association between higher fluid intelligence and stronger proactive control (Burgess and Braver, 2010) as well as between age- and disease-related decline of working memory capacity and diminished proactive control (Paxton et al., 2008; Edwards et al., 2010).

Research on proactive and reactive inhibitory control has so far largely focused on behavioral inhibition [for review see Aron (2011)]. Proactive behavioral inhibition may be triggered by introducing a cue indicating the probability of the occurrence of a stop-signal in a given trial in a stop-signal task or by varying the proportion of stop-signals, resulting in proactive adjustments of the speed/accuracy trade-off and, in turn, in longer reaction times and increased accuracy or improved SSRT (Chikazoe et al., 2009; Verbruggen and Logan, 2009; Zandbelt et al., 2010; Jahfari et al., 2012; Swann et al., 2013). However, the same logic can be applied to paradigms capturing other components of impulse control. Burgess and Braver (2010) for instance varied the proportion of recent negative (interference) trials vs. recent positive (facilitation) trials in a recent probes task to manipulate interference expectancy. In that study, however, behavioral parameters were not significantly modulated by interference expectancy.

## Prefrontal Cortex Functioning Underlying Impulse Control

Impulse control is associated with prefrontal functioning especially in the ventrolateral prefrontal cortex (VLPFC)/inferior frontal gyrus (IFG), the insula, the dorsolateral prefrontal cortex (DLPFC), ventromedial prefrontal cortex (VMPFC), and the rostral and dorsal anterior cingulate cortex (ACC) (Laird et al., 2005; Alvarez and Emory, 2006; Nee et al., 2007; Robbins et al., 2012; Aron et al., 2014). As differential patterns of activation have been demonstrated among different tasks and associated impulse components, it has been suggested that impulse control processes acting upon stimulus encoding, response selection, and response execution may recruit brain regions within this network to differing extents (Nee et al., 2007). Therefore, we will first give a short overview of prefrontal activation patterns associated with the abovementioned components of impulse control in healthy participants before reviewing findings in BPD and ADHD populations.

### Prefrontal cortex functioning underlying components of impulse control

#### Prefrontal activation and stimulus interference

Prefrontal activation underlying stimulus interference as assessed with the Stroop task has consistently been found to be strongly left-lateralized. Clusters of activation have been reported especially in left dorsal prefrontal regions like the DLPFC and inferior frontal junction (IFJ), and also in the VLPFC and insula as well as in medial prefrontal regions including the ACC (Derrfuss et al., 2005; Laird et al., 2005; Nee et al., 2007). In addition, smaller clusters of activation have also been observed in the homologue regions in the right hemisphere (Derrfuss et al., 2005; Laird et al., 2005; Nee et al., 2007).

#### Prefrontal activation and proactive interference

Resolution of proactive interference as captured with recent probes tasks has revealed a central role of the left IFG, especially of the pars triangularis subdivision (Badre, 2005; Jonides and Nee, 2006). In addition, the pars orbitalis subdivision of the left IFG as well as right inferior frontal regions have been implicated to be involved in interference resolution in the recent probes task (Badre, 2005; Oztekin and Badre, 2011). Other tasks capturing proactive interference such as directed forgetting tasks, however, have been associated with right-lateralized activation patterns with clusters of activations in the right IFG and middle frontal gyrus (MFG) (Depue, 2012).

#### Prefrontal activation and response interference and behavioral inhibition

Whereas response interference has been associated with activation in bilateral VLPFC, DLPFC, IFJ, as well as with activation in medial prefrontal regions including the ACC/pre-SMA (Nee et al., 2007; Kim et al., 2012), behavioral inhibition has been shown to rely more strongly on a right-lateralized prefrontal activation pattern (Simmonds et al., 2008; Aron, 2011; Swick et al., 2011). Left VLPFC has also been implicated in behavioral inhibition. However, activity located in the left VLPFC seems to be less pronounced compared to the right VLPFC in behavioral inhibition (Swick et al., 2008; Rodrigo et al., 2014). Although common activation during behavioral inhibition in go/no-go- and stop-signal tasks has been shown in clusters in the right VLPFC, IFJ, and pre-SMA (Rubia et al., 2001; Swick et al., 2011; Sebastian et al., 2013b), increased activation during inhibition in a stop-signal task as compared to the go/no-go task has been reported in the right VLPFC, left insula, and the pre-SMA (Swick et al., 2011; Sebastian et al., 2013b). Activation in the IFJ during behavioral inhibition has rather been linked to attentional processes than to inhibitory functioning (Chikazoe et al., 2008; Verbruggen et al., 2010; Boehler et al., 2011).

#### Prefrontal activation and information sampling

Information sampling has been shown to rely on ventromedial prefrontal regions and the left DLPFC (Heekeren et al., 2008; Basten et al., 2010). The posterior DLPFC has been suggested to not only be involved in computing a decision but also translating it into an action independently of response modality (Heekeren et al., 2008). In addition, the ACC has been shown to index conflict at the decision stage (Pochon et al., 2008).

#### Prefrontal activation and delay discounting

Delay discounting assesses a motivational component of impulse control. A recent meta-analysis revealed bilateral prefrontal activation in the anterior insula, DLPFC, and the ACC with larger clusters of activation in the left hemisphere (Wesley and Bickel, 2014). Brain activity in the VMPFC, especially in the medial OFC, as well as in the ventral striatum has been associated with the subjective value of immediate and delayed outcomes, whereas DLPFC seems to modulate value signals in other regions rather than to contribute to the valuation process *per se* (Kable and Glimcher, 2007; Peters and Büchel, 2011). Brain activation in ACC and lateral PFC has been associated with hard vs. easy choices in delay discounting paradigms (Peters and Büchel, 2011). At least three neural networks have been associated with different aspects of delay discounting: (1) a ventral cortico-striatal network comprising medial OFC and ventral striatum has been associated with individual differences in reward value, i.e., the representation of the incentive value of a broad range of different classes; (2) a lateral prefrontal-cingulate network including lateral OFC, dorsolateral and ventrolateral PFC as well as cingulate cortex has been linked to conflict detection and behavioral inhibition, and (3) a medial temporal-hippocampus network has been implicated in prospective evaluation of future outcomes [for reviews see Peters and Büchel (2011) and Bari and Robbins (2013)].

### Prefrontal cortex functioning underlying proactive and reactive inhibitory control

Several regions within the prefrontal cortex such as the VLPFC, the DLPFC, the IFJ, as well as pre-supplementary and premotor areas were suggested to implement proactive and reactive control modes (Braver et al., 2009; Aron, 2011). This has been shown not only for studies employing modified stop-signal tasks (Chikazoe et al., 2009; Jahfari et al., 2012; Swann et al., 2013), but also for tasks capturing other components of impulse control. By varying the expectancy of interference in a recent probes task, Burgess and Braver (2010) assessed the effect of proactive vs. reactive inhibitory cognitive control. Lateral and prefrontal activation corresponded to reactive cognitive impulse control in the low expectancy condition, as well as to proactive cognitive impulse control in the high expectancy condition. Of note, during cognitive impulse control global sustained activation (i.e., on all trials) of lateral prefrontal areas was observed, suggesting sustained, anticipatory and/or preparatory prefrontal activation. Similarly, Braver et al. (2003) reported left lateral PFC activity associated with both, sustained/proactive and with transient/reactive impulse control in a task-switching paradigm. Jahfari et al. (2012) noted that although both, proactive and reactive impulse control, rely on prefrontal regions (together with basal ganglia), prefrontal activation is strongest during reactive stopping on the one hand whereas proactive impulse control reduces the need for reactive fronto-striatal activation to gate voluntary action.

Thus, while recruiting overlapping neural networks proactive and reactive control differ in the temporal dynamics of prefrontal activity, i.e., proactive control relies on sustained cue-related anticipatory activity whereas reactive control is based on transient probe-related activity (Braver et al., 2009). The diverging temporal dynamics of proactive and reactive impulse control are well compatible with the assumptions of theoretical models of hierarchical rostro-caudal functional specialization within lateral PFC (Koechlin and Summerfield, 2007; Badre and D’Esposito, 2009), in the sense that sustained cue-related activity is predicted to be more prominent in rostral lateral PFC regions, implementing proactive control, e.g., in the DLPFC, whereas transient probe-related activity, related to reactive control, is more likely to occur in caudal PFC regions, such as the VLPFC (Aron, 2011). Furthermore, the neural underpinnings of proactive and reactive inhibitory control might be even better understood from the perspective of the tonic-phasic dopamine hypothesis (Floresco et al., 2003), i.e., “proactive” activity of rostral PFC regions may be regulated by tonic dopaminergic modulation and “reactive” activity of caudal PFC regions by phasic dopaminergic input.

### Summary of prefrontal activation patterns of impulse control

Taken together, components of impulse control have been shown to rely on prefrontal regions including the VLPFC, DLPFC, IFJ, insula, OFC, as well as medial frontal regions such as the VMPFC, the ACC, and the pre-SMA. Whereas behavioral inhibition is associated with a right-lateralized prefrontal network, other impulse control components have been shown to rely on a bilateral (response interference, delay discounting) or rather left-lateralized prefrontal network (stimulus interference, proactive interference, and information sampling). One must note, however, that most of the tasks assessing stimulus interference (e.g., Stroop task), proactive interference (e.g., recent probes), or delay gratification (e.g., delay discounting task) involve verbal material. With respect to proactive and reactive impulse control, it appears likely that proactive control is implemented by more rostral lateral PFC regions such as the DLPFC and reactive control is mediated by more caudal regions of lateral PFC such as the VLPFC.

Given these activation patterns associated with different components of impulse control in healthy subjects we review and systematically compare neurobehavioral findings in BPD and ADHD in the next sections to answer the question to what extent prefrontal dysfunctions are related to distinct disinhibitory or impulse control components. As for disinhibition of proactive and reactive control, no clear statements can be made for BPD and ADHD because, to the best of our knowledge, systematic neuroimaging studies on this topic are not yet available. Some of the available studies have, however, focused on sustained vs. transient impulse control. Therefore, if applicable, these findings will be discussed within the framework of proactive vs. reactive control. We believe that this is an important issue as in theory it is plausible that highly impulsive subjects act less in the proactive impulse control mode since they utilize fewer cues to control their behavior.

## Prefrontal Cortex Functioning Underlying Components of Impulse Control in BPD

Although impulsivity is a clinical, diagnostic, and pathophysiological hallmark of BPD only few neuroimaging studies have investigated disturbed impulse control in patients with BPD. Most of these studies have focused on the emotional modulation of impulse control as emotional dysregulation has been shown to interact with impulse control especially for BPD-salient emotions whereas experimental paradigms assessing emotionally neutral impulse control in BPD have revealed rather weak and inconsistent results [for review see Sebastian et al. (2013a)].

### Prefrontal dysfunctions in BPD associated with stimulus interference

Two studies have assessed neural networks underlying stimulus interference in BPD using fMRI. Disturbances in stimulus interference have previously been implicated in BPD various neuropsychological studies (Ruocco, 2005). Wingenfeld et al. (2009) used an emotional Stroop task including neutral words, general negative words, and individual negative words in a block design, i.e., three blocks for each word category. Participants were required to name the colors in which the words were printed. Whereas healthy control participants displayed increased activation in prefrontal regions comprising the dorsal and rostral parts of the ACC and the medial frontal cortex during general negative as compared to neutral words, patients with BPD did not display corresponding signal changes. Similarly, when comparing individual negative words to neutral words only healthy controls showed increased activation in the ACC and the right OFC. When directly comparing both groups patients with BPD accordingly displayed decreased activation in fronto-limbic regions including the medial frontal gyrus and dorsal ACC during generally and individually emotionally modulated resolution of stimulus interference, respectively. While the dorsal part of the ACC has been associated with cognitive functions such as modulation of attention, executive functions, complex motor control, and the rostral ACC has been implicated in emotion regulation (Bush et al., 2000). The medial prefrontal gyrus is important for both emotion and stress regulation (Davidson, 2002). A recent meta-analysis has revealed relative hypoactivation of subgenual and dorsal ACC in patients with BPD associated with negative emotionality (Ruocco et al., 2013). Thus, during emotionally modulated stimulus interference, patients with BPD failed to activate the ACC and medial frontal brain regions, which are essential for the regulation of emotions and stress (Wingenfeld et al., 2009) supporting the notion that regulatory processes of negative emotions are deficient in BPD.

Holtmann et al. (2013) used a modified Flanker task (Eriksen and Eriksen, 1974) with task-irrelevant neutral and emotional, i.e., fearful faces as distracters displayed in the background during event-related fMRI. In this paradigm, a central arrowhead, pointing either to the right or left, is flanked by four surrounding arrowheads pointing either in the same (congruent condition) or opposite direction (incongruent condition) of the central arrowhead. In the incongruent condition, interference arises, which has to be inhibited. The Flanker task thus captures distractor- and response-related interference (Stahl et al., 2014). Both, patients with BPD and healthy control subjects, displayed longer reaction times in the incongruent as compared to the congruent condition, longer reaction times during emotional as compared to neutral conditions, as well as an emotion by congruency interaction with longest reaction times in emotional incongruent trials. Yet, no group effect was observed on a behavioral level. Whole brain imaging results revealed no group differences in activation of prefrontal regions for the congruency effect. Region of interest analysis resulted in activation in the DLPFC and, similar to the findings of Wingenfeld et al. (2009), in the dorsal ACC in healthy control subjects only when comparing successful incongruent and congruent trials. Interaction of interference inhibition with emotion was associated with increased activation in the right amygdala and the intra-parietal sulcus in patients, but not in healthy control participants. No interaction effect was observed in prefrontal regions.

**Figure 1:**
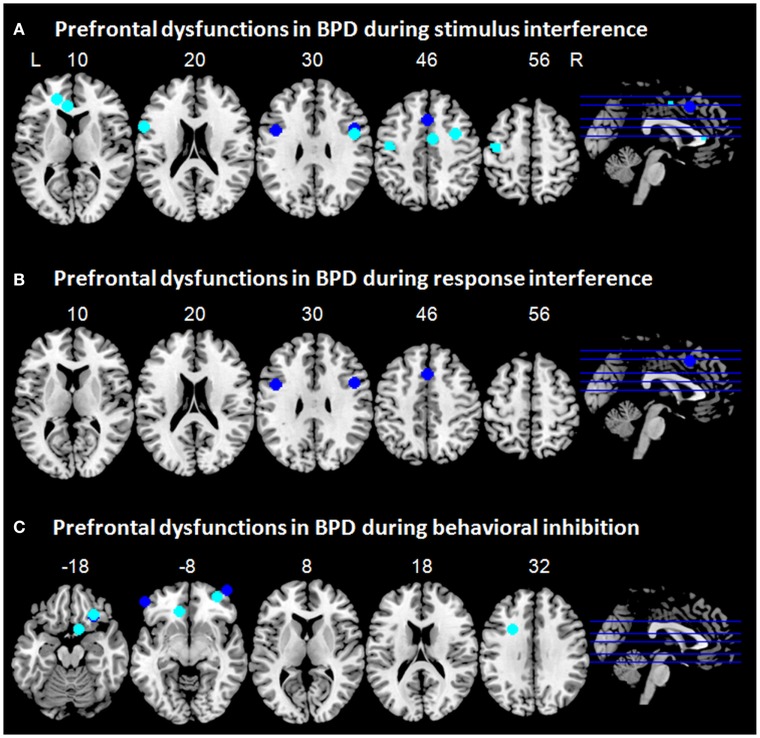
**Prefrontal dysfunctions in borderline personality disorder (BPD)**. Maxima of clusters of prefrontal dysfunctions during **(A)** stimulus interference, **(B)** response interference, or **(C)** behavioral inhibition are displayed as reported by Holtmann et al. (2013), Jacob et al. (2013), Silbersweig et al. (2007), and Wingenfeld et al. (2009). Blue, prefrontal dysfunctions associated with emotionally neutral impulse control; cyan, prefrontal dysfunctions associated with emotionally modulated impulse control. L = left; R = right.

Taken together, while patients with BPD did not differ behaviorally from healthy controls, stimulus interference in BPD might be associated with hypoactivation in the ACC, especially in the dorsal, cognitive portion of the ACC (Bush et al., 2000). As ACC dysfunction was revealed during blocked as well as during event-related fMRI, one might speculate that this might subserve sustained or proactive as well as transient or reactive stimulus interference. DLPFC hypofunction was linked to stimulus interference only during event-related fMRI and might therefore rather be involved in reactive stimulus interference. During emotionally modulated stimulus interference, patients with BPD displayed hypoactivation in neural networks typically associated with emotion regulation such as ACC and DLPFC, which have been shown to be less activated in patients with BPD during negative emotionality (Ruocco et al., 2013). Hence, these patterns of hypoactivity in BPD might resemble rather dysfunctional processing of negative emotional stimuli than disturbances associated with stimulus interference *per se*. One must note, however, that only very few imaging studies have so far assessed stimulus interference in BPD and no imaging studies could be identified that studied stimulus interference in a pure emotionally neutral setting in BPD. Hence, we can interpret these preliminary findings only cautiously. To illustrate prefrontal dysfunctions in patients with BPD during stimulus interference, maxima of clusters as reported in the above mentioned studies are displayed in Figure 1A.

### Prefrontal dysfunctions in BPD associated with proactive interference

Proactive interference has barely been studied up to now in BPD. Only one study so far has used a recent probes task (Krause-Utz et al., 2012). In that study, however, rather the effect of emotional distractors on working memory performance was assessed than resolution of proactive interference. Patients with BPD showed significantly longer reaction times along with significantly higher activation in the amygdala and insula during emotional distraction as compared to healthy participants, whereas during neutral control conditions no behavioral group differences were observed. The authors concluded that hyper-responsiveness to emotionally distracting pictures negatively affects working memory performance in patients with BPD. One must note, however, that the authors focused on working memory performance (using a modified Sternberg item recognition task) and not on resolution of proactive interference in that study, i.e., they did not assess interference of contents of memory sets from previous trials on the current trials. Although behavioral studies using directed forgetting paradigms indicate dysfunctional proactive inhibition in BPD (Korfine and Hooley, 2000; Domes et al., 2006), no brain imaging studies assessing differences in brain activation patterns associated with proactive interference in BPD could be identified. This holds true for a recent fMRI study of Prehn et al. (2013) testing working memory – emotion interaction in a sample of male antisocial personality disorder (ASPD) and patients with BPD. During emotionally neutral working memory, ASPD–BPD subjects did not differ in general task performance and neural representation of working memory processes from comparison subjects. When the memory task was combined with emotional background pictures ASPD–BPD subjects showed delayed responses and enhanced activation of the left amygdala in the presence of emotionally high salient pictures independent of working memory load (Prehn et al., 2013).

### Prefrontal dysfunctions in BPD associated with response interference

No neuroimaging studies in BPD using classical paradigms such as task switching or response priming tasks are to date available, which assess resolution of response interference (Klauer et al., 2005). Only one study used a flanker task (Holtmann et al., 2013), which allows assessing aspects of stimulus interference and response interference (Stahl et al., 2014) and which has been reported above. Figure 1B illustrates maxima of clusters of prefrontal dysfunctions in patients with BPD during response interference as reported by Holtmann et al. (2013).

### Prefrontal dysfunctions in BPD associated with behavioral inhibition

The great majority of studies using behavioral inhibition tasks such as go/no-go or stop-signal tasks did fail to reveal performance deficits as this should be indicated by increased commission error rates in go/no-go tasks or by increased stop-signal reaction time in stop-signal tasks in patients with BPD [Nigg et al., 2005; Lampe et al., 2007; Ruchsow et al., 2008; Völker et al., 2009; Jacob et al., 2010; LeGris et al., 2012; Hagenhoff et al., 2013; but see Ruocco et al. (2012) for deficits in patients with BPD in a continuous performance tasks measuring response inhibition, vigilance, and sustained attention]. This suggests that patients with BPD do not display behavioral deficits in behavioral inhibition as captured with neutral response inhibition tasks [for a review see Sebastian et al. (2013a)], at least under baseline non-stressed, non-emotional conditions (Krause-Utz et al., 2013; Cackowski et al., 2014).

Accordingly, findings from fMRI studies have revealed – if at all – only subtle differences in activation patterns in patients with BPD associated with behavioral inhibition. One must note, however, that up to now only few neuroimaging studies have assessed emotionally neutral behavioral inhibition in BPD. In the fMRI study by Jacob et al. (2013), individuals with BPD and healthy control participants performed a go/no-go paradigm in a block design after induction of anger, joy, or a neutral mood. Patients neither differed in their behavioral performance nor in brain activation patterns associated with behavioral inhibition for emotionally neutral contexts. Silbersweig et al. (2007) used a verbal go/no-go task, which comprised neutral, positive, or BPD-specific negative stimuli in a block design. Only subtle group differences in brain activation patterns were found during behavioral inhibition in the neutral condition. Prefrontal dysfunctions in the BPD group comprised relatively decreased activation in bilateral OFC. However, no differences were found in key regions of the neural behavioral inhibition network, such as the right VLPFC or pre-SMA.

Whereas neutral behavioral inhibition reveals only subtle prefrontal dysfunctions in BPD this picture changes substantially when emotions come into play. After induction of anger, patients with BPD as compared to healthy control participants showed decreased activation in the left IFG during behavioral inhibition, which was accompanied by increased activation of the subthalamic nucleus (STN) (Jacob et al., 2013). Since a hyperdirect pathway from the lateral prefrontal cortex via the STN has been described for effective response inhibition (Aron and Poldrack, 2006; Aron et al., 2007), this might be interpreted as a compensatory mechanism for reduced prefrontal activation. According to Jacob et al. (2013) this might explain why patients with BPD often do not show impaired performance in behavioral inhibition tasks, even if emotional stimulus material is used. In the study by Silbersweig et al. (2007), the interaction of BPD-related negative emotion and behavioral inhibition revealed prefrontal dysfunctions in BPD, i.e., decreased activity in the VMPFC including medial OFC and subgenual ACC in concert with relative hyperactivation in right lateral OFC/VLPFC and left DLPFC. In addition, decreased VMPFC activation was highly correlated with negative emotion. Of note, prefrontal regions involved in cognitive emotion regulation as well as in behavioral inhibition such as ventrolateral OFC/PFC and dorsal ACC, showed increased activity potentially trying to compensate for frontolimbic dysfunctions. To illustrate prefrontal dysfunctions in patients with BPD associated with response inhibition, maxima of clusters as reported in the above mentioned studies are displayed in Figure 1C.

Both studies on behavioral inhibition in BPD used a block design, i.e., the go/no-go task contained go-blocks comprising only go trials and no-go blocks comprising about 40% no-go trials, which were contrasted to assess neural correlates underlying behavioral inhibition (Silbersweig et al., 2007; Jacob et al., 2013). As the expectancy of no-go trials is higher in no-go as compared to go blocks, contrasting both conditions should reveal mainly brain activation associated with proactive behavioral inhibition. Taken together, the few fMRI studies focusing on proactive behavioral inhibition in BPD have revealed prefrontal dysfunctions especially if modulated by negative emotions. Evidence from neuroimaging studies in concert with behavioral studies suggests, however, that behavioral inhibition is largely intact in BPD, at least under emotionally neutral, non-stressed conditions.

### Prefrontal dysfunctions in BPD associated with information sampling

Evidence from behavioral studies suggests that individuals with BPD display risky decision making even if constantly provided with feedback regarding the consequences of the decision (Svaldi et al., 2012). In the study by Cackowski et al. (2014), no significant effect of stress on risky decision making (as assessed by the IOWA gambling task) was observed, whereas stop-signal task performance was significantly impaired after a stress induction in patients with BPD. It has been assumed that risky decision making in BPD may result from deficits in integrating reinforcement signals during decision making opting for risky choices even if clearly avoidable (Kirkpatrick et al., 2007). Accordingly, patients with a cluster B personality disorder unlike healthy control subjects did not show activation in lateral and medial prefrontal brain regions during reinforcement processing, which may underlie some of the deficits in decisional impulse control observed in these patients (Völlm et al., 2007). In sum, these findings provide a preliminary indication that prefrontal hypofunction underlie decisional impulsivity in BPD, which remains to be verified in future neuroimaging studies.

### Prefrontal dysfunctions in BPD associated with delayed discounting

Only few studies up to now have assessed delay discounting in BPD. Two of these studies resulted in increased preference for immediate over delayed reward in patients with BPD (Völker et al., 2009; Lawrence et al., 2010). Coffey et al. (2011) however, report increased preference for immediate over delayed reward only in patients with BPD with current or past substance use disorder, but not in patients with BPD without substance abuse. In contrast to other components of impulse control, deficient information sampling and delay discounting do not appear to be modulated by negative emotions in BPD (Lawrence et al., 2010; Cackowski et al., 2014).

Although behavioral findings strongly suggest deficient delay discounting in BPD, hitherto no neuroimaging study has assessed neural correlates associated with classical delay discounting tasks in BPD. Völlm et al. (2007) studied neural correlates of reward and loss in a small group of patients (*N* = 8) with cluster B personality disorders, i.e., BPD and antisocial personality disorder. Group comparisons during reward revealed prefrontal hypoactivation in the patients in left medial OFC, left DLPFC, right frontal pole, as well as in ACC, whereas hyperactivation was present in the bilateral medial frontal cortex extending to amygdala. Loss was associated with prefrontal hypoactivation in the patients in bilateral DLPFC, whereas hyperactivation was observed in bilateral medial PFC, left MFG, as well as in the ACC. As DLPFC and ACC comprise prefrontal regions of the neural network underlying delay discounting, these regions might be candidate regions for deficient delay discounting in BPD.

### Summary of prefrontal dysfunctions in BPD

In BPD, both stimulus interference and response interference have been associated with hypoactivation in the dorsal, cognitive portion of the ACC, and the DLPFC (Wingenfeld et al., 2009; Holtmann et al., 2013; Figure 1). These regions have also been implicated in negative emotionality in BPD (Ruocco et al., 2013). As the paradigms that have been used to assess stimulus and response interference in BPD comprised emotional material, it remains to be tested whether the observed hypoactivation rather underlies disturbed emotion processing or whether it can directly be attributed to impulse control deficits in BPD. During behavioral inhibition, patients with BPD have been shown to exhibit medial prefrontal hypoactivation mainly in orbitofrontal regions (Silbersweig et al., 2007; Jacob et al., 2013). Although studies assessing behavioral inhibition in patients with BPD have mainly focused on emotional modulation of behavioral inhibition, and medial frontal dysfunction in BPD has been implicated in emotional dysregulation (Kamphausen et al., 2013; Ruocco et al., 2013; Krause-Utz et al., 2014), medial prefrontal dysfunction was also present in neutral conditions of behavioral inhibition (Silbersweig et al., 2007). No neuroimaging studies could be identified that directly assessed neural correlates of proactive interference, information sampling, or delay discounting. One study focused on reward and loss processing in a small group of patients with different cluster B personality disorders including BPD and revealed that reward and loss processing in that group was associated with dysfunction in medial, orbitofrontal, and dorsolateral prefrontal regions (Völlm et al., 2007). This might suggest that dysfunctions in these regions might subserve deficient delay discounting in BPD. One must note, however, that in that study not only patients with BPD, but also patients with other cluster B personality disorder diagnoses were included. Therefore, the results may not be specific to BPD.

## Prefrontal Cortex Functioning Underlying Components of Impulse Control in ADHD

Neuropsychological deficits in executive functions in children with ADHD have been shown to persist into adulthood, with the most consistent findings showing abnormalities in stimulus interference, response interference, and behavioral inhibition. These deficits have most consistently been linked to prefrontal dysfunctions especially in lateral prefrontal regions and the ACC (Cubillo and Rubia, 2010; Hart et al., 2013; Volkow and Swanson, 2013). We will focus in the following sections on adult ADHD but we will also consider findings from childhood ADHD whenever no or too little studies on adult ADHD are available.

### Prefrontal dysfunctions in ADHD associated with stimulus interference

Patients with ADHD display increased stimulus interference as captured by Stroop tasks (Lansbergen et al., 2007). Two fMRI studies have assessed neural correlates of deficient stimulus interference in adult ADHD. Banich et al. (2009) employed a mixed design during a classical computerized color-word Stroop task. Event-related analysis resulted in hypoactivation in the right VLPFC and ACC in patients with ADHD when contrasting incongruent to neutral trials. When comparing incongruent blocks to congruent or neutral blocks, patients with ADHD exhibited hyperactivation in the right DLPFC. The authors suggest that DLPFC hyperactivity resulting from the block-wise analysis might reflect top-down biasing of sustained attention in ADHD, whereas hypoactivation in the right VLPFC and ACC might rather reflect dysregulation of the resolution of stimulus interference at a transient, reactive attentional level. Error trials were excluded from the event-related analysis only. Therefore, differences in activation patterns may not only rely on differences in transient and sustained attention but also in differences in error processing (which is a general drawback of blocked designs in impulse control research). However, regions implicated in error processing are rather anterior insula and dorsal ACC (Aron and Poldrack, 2006; Agam et al., 2014; Erika-Florence et al., 2014; Steele et al., 2014) than the DLPFC, which in turn has been implicated in working memory performance (Brunoni and Vanderhasselt, 2014; Caspers et al., 2014) as well as in proactive inhibition (Chikazoe et al., 2009; Aron, 2011; Jahfari et al., 2012). Increased DLPFC activity in patients with ADHD resulting from the block-wise analysis might hence indicate increased working memory demands or proactive stimulus interference.

Hypofunction of dorsal ACC has also been shown in ADHD during a counting Stroop task in a blocked design (Bush et al., 1999). In addition, patients with ADHD in that study exhibited hypofunction in the left DLPFC, whereas prefrontal hyperfunction was observed in bilateral VLPFC and insula. Although the finding of relative DLPFC hypofunction in patients with ADHD in a blocked Stroop task is at contrast to the findings by Banich et al. (2009), both studies suggest dysfunctions in neural networks subserving proactive, sustained stimulus interference potentially in concert with increased working memory demands. This notion is supported by a recent meta-analysis on interference inhibition and attention in pediatric and adult samples of patients with ADHD. The meta-analysis revealed hypoactivation in the right VLPFC/insula and in the dorsal ACC in patients with ADHD during interference inhibition (Hart et al., 2013). However, interference inhibition tasks in this meta-analysis comprised paradigms, which are associated with stimulus interference (i.e., Stroop task), response interference (i.e., Simon task), or both (i.e., Flanker task). Thus, the dysfunctions presented in that study are not specific to stimulus interference but may subserve also deficient response interference in ADHD.

**Figure 2:**
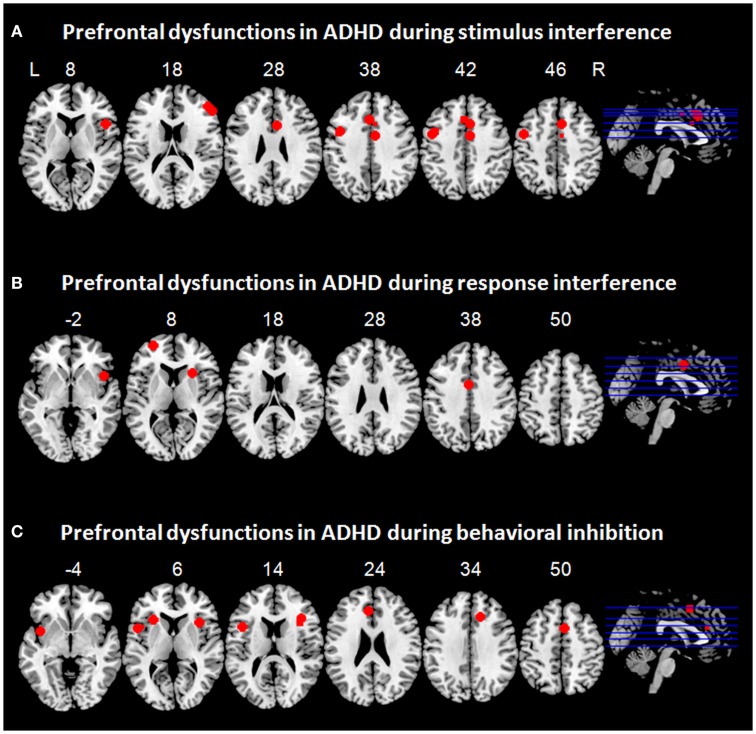
**Prefrontal dysfunctions in attention-deficit/hyperactivity disorder (ADHD)**. Maxima of clusters of prefrontal dysfunctions during **(A)** stimulus interference, **(B)** response interference, or **(C)** behavioral inhibition are displayed as reported by Banich et al. (2009), Burgess and Braver (2010), Bush et al. (1999), Cubillo et al. (2010, 2011), Epstein et al. (2007), Hart et al. (2013), and Sebastian et al. (2012). Blue, prefrontal dysfunctions associated with emotionally neutral impulse control. L = left; R = right.

Taken together, evidence from two fMRI studies suggests frontal dysfunctions in ventrolateral and dorsolateral PFC as well as in the dorsal ACC during resolution of stimulus interference in adult patients with ADHD. DLPFC dysfunction can most likely be linked to sustained, proactive stimulus interference in ADHD, whereas VLPFC dysfunction might rather underlie transient, reactive stimulus interference. To illustrate prefrontal dysfunctions in patients with ADHD associated with stimulus interference, maxima of clusters as reported in the above mentioned studies are displayed in Figure 2A.

### Prefrontal dysfunctions in ADHD associated with proactive interference

No neuroimaging studies could be identified that have been studying proactive interference in ADHD. Two studies have used a Sternberg paradigm (Wong and Stevens, 2012; Lenartowicz et al., 2014). However, these studies rather focused on working memory impairments in ADHD than on resolution of proactive interference. The critical contrast of non-recent as compared to recent target capturing proactive interference was not assessed in those studies. As patients with ADHD have been reported to show dysfunctions in a wide range of impulse control processes, studies on proactive interference and underlying neural networks in ADHD are necessary.

### Prefrontal dysfunctions in ADHD associated with response interference

Evidence from studies in childhood ADHD suggests hypoactivation in bilateral VLPFC and insula during switch tasks (Smith et al., 2006; Rubia et al., 2009b) and hypoactivation in medial prefrontal regions, i.e., ACC, during Simon tasks (Rubia et al., 2011). Similarly, response interference during a switch task in adults ADHD was associated with prefrontal hypoactivation in bilateral VLPFC and insula (Cubillo et al., 2010). During successful resolution of response interference in a Simon task, adult patients with ADHD exhibited hypoactivation not only in lateral prefrontal regions such as VLPFC and insula, but also in the OFC and cingulate regions (Cubillo et al., 2011; Sebastian et al., 2012). Recently, a meta-analysis was performed on tasks capturing different aspects of interference inhibition such as response interference and stimulus interference (Hart et al., 2013). This resulted in prefrontal hypoactivation in the right VLPFC/insula and in the dorsal ACC (Hart et al., 2013). It remains to be tested whether hypoactivation in certain regions corresponded more strongly to one of these two components of impulse control. To illustrate prefrontal dysfunctions in patients with ADHD associated with response interference, maxima of clusters as reported in the above mentioned studies are displayed in Figure 2B.

### Prefrontal dysfunctions in ADHD associated with behavioral inhibition

Whereas a vast functional imaging literature in childhood ADHD on behavioral inhibition exists, only few studies hitherto have employed go/no-go and stop-signal tasks in adult patients with ADHD. These studies have quite consistently revealed hypofunction in a fronto-striatal network in patients with ADHD during successful behavioral inhibition with prefrontal dysfunction comprising VLPFC and insula (Epstein et al., 2007; Cubillo et al., 2010; Sebastian et al., 2012). Two studies failed to show VLPFC hypofunction in adult ADHD (Dibbets et al., 2009; Carmona et al., 2012). Yet, a meta-analysis on go/no-go and stop-signal tasks in childhood and adult ADHD revealed prefrontal hypofunction in right VLPFC/insula, ACC, and SMA, along with subcortical hypofunction in striatum (Hart et al., 2013). Moreover, a categorical comparison of childhood vs. adult ADHD implicated that hypofunction in ACC/SMA and basal ganglia was more pronounced in childhood ADHD, whereas right VLPFC deficiency was more prominently associated with adult ADHD. The authors suggested that frontal deficits may become more prominent with age and may be secondary to primary subcortical deficits, which may normalize in adult ADHD (Hart et al., 2013). Findings regarding ACC dysfunction are inconsistent, potentially as a function of the paradigm employed: Whereas Epstein et al. (2007) reported hyperfunction of ACC during behavioral inhibition in a go/no-go task, Cubillo et al. (2010) reported ACC hypofunction during behavioral inhibition in a stop-signal task. Taken together, findings from neuroimaging studies on behavioral inhibition in adult ADHD converge in VLPFC and insula hypofunction accompanied by striatal hypofunction and disturbed ACC activity. To illustrate prefrontal dysfunctions in patients with ADHD associated with behavioral inhibition, maxima of clusters as reported in the above mentioned studies are displayed in Figure 2C.

### Prefrontal dysfunctions in ADHD associated with information sampling

Studies assessing impulsive decision making in ADHD have largely used gambling and risk-taking paradigms. Poor decision making and inappropriate risk taking has been shown to reflect problems in both analytic/deliberate and affective neurocognitive systems (Mantyla et al., 2012). With respect to gambling behavior, ADHD symptoms have been shown to correlate with self-reported gambling behavior as well as performance in a computer-based gambling task (Dai et al., 2013). Regarding decision making, Mantyla et al. (2012) suggest that ADHD is associated with impaired decision making in tasks involving a significant degree of cognitive control and prefrontally mediated executive functions.

Only two neuroimaging studies could be identified that assessed impulsive decision making in adult ADHD. Wilbertz et al. (2012) correlated gambling behavior with altered medial OFC activity in patients with ADHD underlying insensitivity to the motivational value of outcomes. Thereby, dysfunctional incentive modulation of OFC activity was associated with more risky decisions and insufficient feedback processing in the gambling task. The authors concluded that this might reflect insensitivity to negative consequences of risky behavior. Ibanez et al. (2012) assessed event-related potentials (ERP) during gambling tasks in patients with ADHD. Compared to healthy controls, patients with ADHD exhibited deficient error-related negativity, i.e., no effect of valence (win or loss), implicating impaired learning by feedback. Source localization revealed that ERP findings were associated with hypoactivation in cingulate regions including the ACC. In sum, both studies have linked medial prefrontal hypoactivation to impulsive decision making in adult ADHD. One must note, however, that none of these studies directly assessed neural correlates of impulsive decision making. In the study by Wilbertz et al. (2012), the gambling task was performed outside the scanner. Performance parameters were than correlated with brain imaging results obtained from a different paradigm. In the study by Ibanez et al. (2012), no behavioral between-group differences were observed for decision-making under risk or ambiguity. Whether or not differences in neural underpinnings of risky decision making were present was not directly assessed. Rather, error-related negativity (i.e., effect of valence) was studied. Subsequent source modeling was restricted to regions of interest within the cingulate cortex. Therefore, these studies provide only indirect and preliminary evidence that deficient information sampling and impulsive decision making in ADHD might be associated with disturbed activation in medial prefrontal regions. Yet, studies directly addressing that question are lacking to date.

### Prefrontal dysfunctions in ADHD associated with delayed discounting

Delay-related impulsivity or a preference for smaller, immediate rewards over larger, delayed rewards has been implicated in etiological models of ADHD which either focus on delay aversion (Sonuga-Barke, 2005) or on the role of dopamine-mediated learning processes (Sagvolden et al., 2005). Accordingly, delay-related impulsivity has been shown for children and adolescents (Paloyelis et al., 2010; Demurie et al., 2012; Scheres et al., 2013) as well as for adults with ADHD (Hurst et al., 2011; Dai et al., 2013). Steep discouting has thereby been rather associated with symptoms of impulsivity and hyperactivity than with inattention (Scheres et al., 2008, 2010, 2013). Of note, in some studies adult patients with ADHD did not differ in discounting rates from healthy controls (Wilbertz et al., 2012, 2013), whereas others suggested that steeper discounting is confined to patients with ADHD with concurrent substance dependency (Crunelle et al., 2013). However, in the study by Crunelle et al. (2013) the proportion of patients with inattentive subtype was considerably higher in the ADHD only group which might have influenced the results.

Recently, three distinct brain networks have been suggested that could be implicated in ADHD, especially with respect to different aspects of delay-related impulsivity and decision making: (1) deficits in goal setting and implementation of intention might result from altered connectivity patterns within the default mode network; (2) deficits in a dorsal fronto-striatal network may result in executive dysfunction-mediated impairments in the ability to compare outcome options and make choices; and (3) dopaminergic dysregulation in a ventral fronto-striatal network may disturb processing of cues of future utility, evaluation of experienced outcomes, and learning of associations between cues [for review see Sonuga-Barke and Fairchild (2012)].

Given the broad evidence of delay-related impulsivity in ADHD, it is surprising that hitherto only few neuroimaging studies have been conducted to study alterations or neural underpinnings in adult ADHD. Most of the neuroimaging studies have rather focused on reward anticipation and reward processing (e.g., Ströhle et al., 2008; Stoy et al., 2011; Carmona et al., 2012; Wilbertz et al., 2012; Edel et al., 2013; Furukawa et al., 2014; Plichta and Scheres, 2014). Rubia et al. (2009b) assessed delay discounting in adolescent boys with ADHD (combined subtype). Compared to healthy controls, patients with ADHD exhibited hypoactivation in the right DLPFC and in left prefrontal regions covering OFC, VLPFC, and DLPFC when contrasting delayed to immediate decisions. In addition, increased functional connectivity of left anterior and ventromedial PFC with nucleus accumbens has been associated with delay-related impulsivity in childhood ADHD (Costa Dias et al., 2013). In the study by Plichta et al. (2009) adult patients with ADHD as compared to healthy controls displayed hypoactivation in the ventral striatum toward immediate reward which attenuated in a gradient-like manner towards the dorsal portion of the striatum. By contrast, delayed rewards were associated with hyperactivation in the dorsal striatum which attenuated toward ventral direction. No differences in prefrontal regions were reported.

The ventrolateral deficits in these studies might most likely reflect difficulties in learning the economic significance of cues predicting future reinforcement as interrelated subprocesses such as encoding cue salience and valence, evaluating experienced outcomes, and learning from experience are subserved by ventral fronto-striatal networks. These networks have been shown to link orbitofrontal and VMPFC with ventral striatum and amygdalae. Dorsolateral deficits implicate rather deficits in cognitive or executive functions involved in discounting such as deliberative processes involved in the comparison of choice options (Sonuga-Barke and Fairchild, 2012). These processes will most likely include working memory processes, e.g., by holding choice alternatives in mind, which are linked to DLPFC function (Brunoni and Vanderhasselt, 2014; Caspers et al., 2014). Similarly, DLPFC dysfunctions in ADHD have been implicated stimulus interference, especially in block-wise analysis. Thus, DLPFC dysfunctions across different impulse control components might be linked rather to sustained task demands including working memory processes than to transient demands reflecting reactive inhibitory functioning.

### Summary of prefrontal dysfunctions in ADHD

Stimulus interference in ADHD has been linked to disturbed activation in ACC, DLPFC, and VLPFC (Bush et al., 1999; Banich et al., 2009). Similarly, patients with ADHD exhibit hypofunction of medial prefrontal regions and ventrolateral regions during response interference (Cubillo et al., 2010, 2011; Rubia et al., 2011; Sebastian et al., 2012; Hart et al., 2013). During behavioral inhibition, prefrontal dysfunction in patients with ADHD has mainly been associated with hypoactivation in bilateral PFC (Epstein et al., 2007; Cubillo et al., 2010; Sebastian et al., 2012). Preliminary results suggest that impulsive decision making in ADHD as assessed with gambling tasks and risky choice paradigms may be associated with OFC hypofunction (Wilbertz et al., 2012). However, neuroimaging studies directly assessing information sampling as well as proactive inhibition are lacking. Delay discounting has been related to prefrontal hypofunction in a network comprising ventrolateral and dorsolateral PFC, OFC, and VMPFC (Rubia et al., 2009a; Costa Dias et al., 2013) (Figure 2). Across different impulse control components, ventrolateral prefrontal dysfunctions may rather be linked to deficient transient, reactive inhibitory processes whereas dorsolateral prefrontal dysfunction may be associated with disturbed sustained task demands including proactive inhibition and working memory demands in ADHD.

## Comparison of Prefrontal Dyscontrol in BPD and ADHD Cortex Functioning Underlying Components of Impulse Control

This review evaluated prefrontal dysfunctions in BPD and ADHD with respect to distinct components of impulse control, as recent findings from behavioral and cognitive neuroscience studies suggest related but dissociable components of impulse control along several functional domains. Therefore, neuroimaging studies assessing stimulus interference, proactive interference, response interference, behavioral inhibition, information sampling/impulsive decision making, and delay discounting in BPD and adult ADHD were reviewed.

**Figure 3:**
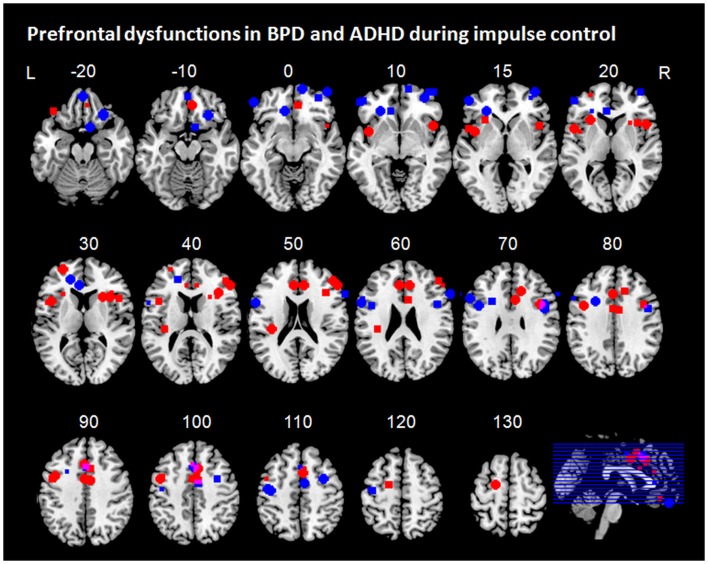
**Prefrontal dysfunctions in borderline personality disorder (BPD) and attention-deficit/hyperactivity disorder (ADHD)**. Maxima of clusters of prefrontal dysfunctions during five components of impulse control (stimulus interference, response interference, behavioral inhibition, risky decision making, and delay discounting) are displayed as reported by Banich et al. (2009), Burgess and Braver (2010), Bush et al. (1999), Cubillo et al. (2010, 2011), Epstein et al. (2007), Hart et al. (2013), Holtmann et al. (2013), Jacob et al. (2013), Rubia et al. (2009b), Sebastian et al. (2012), Silbersweig et al. (2007), Wilbertz et al. (2012), Wingenfeld et al. (2009), and Völlm et al. (2007). Blue, prefrontal dysfunctions associated with impulse control in ADHD; red, prefrontal dysfunctions associated with impulse control in BPD; pink, overlap of ADHD and BPD. L = left; R = right.

Across all components of impulse control, individuals with BPD exhibited frontal dysfunctions mainly in orbitofrontal, dorsomedial (dorsal ACC), and dorsolateral prefrontal regions, whereas individuals with ADHD displayed dysfunctional activation rather in ventrolateral prefrontal regions including IFG and insula, as well as in more dorsal medial frontal regions, particularly in ACC (Figure 3). DLPFC dysfunctions in ADHD seem to be mainly associated sustained task demands such as proactive inhibition and working memory demands. Yet, this overall pattern does not apply to all impulse control components when considering the components separately.

Stimulus interference has been associated with disturbed activation in DLPFC and ACC in both groups (Bush et al., 1999; Banich et al., 2009; Wingenfeld et al., 2009; Hart et al., 2013; Holtmann et al., 2013). In addition, patients with ADHD have been shown to exhibit VLPFC hypofunction (Banich et al., 2009; Hart et al., 2013). As the neural underpinnings of stimulus interference have been shown to comprise a prefrontal network of dorsolateral and ventrolateral prefrontal regions and ACC, frontal dysfunctions in both groups were observed in a network typically associated with stimulus interference. Similarly, both patients with ADHD and BPD have been shown to display frontal dysfunction in expected regions of the neural network associated with response interference. Both groups displayed hypofunction of medial prefrontal regions, which was located more anteriorly in patients with BPD compared to patients with ADHD (Rubia et al., 2011; Sebastian et al., 2012; Hart et al., 2013; Holtmann et al., 2013). While both patients with ADHD and BPD displayed overlapping dysfunctions in medial parts of the network subserving response interference, differential lateral prefrontal dysfunctions have been observed in both groups; whereas patients with BPD showed disturbed activation in the dorsal portion of the lateral PFC (Holtmann et al., 2013), studies in patients with ADHD have revealed additional hypofunction in ventrolateral regions (Cubillo et al., 2010, 2011; Hart et al., 2013). For behavioral inhibition, only patients with ADHD exhibited prefrontal hypofunction in brain regions of the neural network typically associated with that particular impulse control component, i.e., in bilateral PFC, and additionally in the ACC (Epstein et al., 2007; Cubillo et al., 2010; Sebastian et al., 2012). In BPD, however, behavioral inhibition was associated with medial prefrontal hypoactivation mainly in orbitofrontal regions (Silbersweig et al., 2007; Jacob et al., 2013). Such medial prefrontal regions are not typically activated during behavioral inhibition (Aron, 2011) but rather during emotion processing (Phan et al., 2004). In addition, medial prefrontal dysregulation has been implicated in emotional dysregulation and emotion processing in BPD (Ruocco et al., 2013; Krause-Utz et al., 2014). Yet, medial prefrontal hypofunction in BPD was observed even in neutral conditions of behavioral inhibition (Silbersweig et al., 2007). As valence ratings of patients with BPD differed from those of the control group not only with respect to negative but also to neutral conditions, one might speculate that some of the group differences might partly depend on differences in emotion dysregulation even in neutral blocks.

A summary of the findings regarding the remaining impulse control components must remain open. Imaging studies assessing neural correlates of proactive interference could neither be identified for ADHD nor for patients with BPD. Although some studies employed recent probes paradigms, which can be used to study resolution of proactive interference, these studies focused instead on working memory processes. Therefore, neuroimaging studies assessing neural correlates of proactive interference using recent probes or directed forgetting paradigms in patients with ADHD and BPD populations are necessary.

Information sampling in particular has not been studied in ADHD and BPD. Rather, more general impulsive decision making has been investigated using gambling tasks and risky choice paradigms. These revealed hypofunction in orbitofrontal regions in both groups and in bilateral DLPFC in patients with BPD (Völlm et al., 2007; Wilbertz et al., 2012). One must note, however, that in the study by Völlm et al. (2007) in which a reward/loss task was used not only patients with BPD, but also patients with other cluster B personality disorder diagnoses were included. Moreover, the sample assessed was rather small with eight patients in total. Therefore, the findings need to be interpreted with caution and the results may not be specific to BPD. The study by Wilbertz et al. (2012) did not employ a gambling task during fMRI. Instead, scores from a behavioral gambling task were correlated with brain activation in regions of interest during reward delivery. The findings are therefore only indirectly and preliminarily indicative of orbitofrontal dysfunction in ADHD during impulsive decision making. Although indirect evidence of prefrontal dysfunction exist, neuroimaging studies directly assessing neural underpinnings and their alterations in information sampling in ADHD and BPD are largely lacking. The same applies to delay discounting. In ADHD, delay discounting has been related to prefrontal hypofunction in a network comprising ventrolateral and dorsolateral PFC, OFC, and VMPFC indicative of deficient reinforcement learning and cognitive subprocesses delay of gratification (Rubia et al., 2009a; Costa Dias et al., 2013). Neuroimaging studies assessing delay of gratification in BPD are, however, lacking. Based on one study in a small group of patients with cluster B personality disorders focusing on processing of reward and loss, one might speculate that similar to patients with ADHD individuals with BPD might most likely display dysfunctions in two networks subserving subporcesses of delay discounting: in a dorsal fronto-striatal network subserving executive dysfunction-mediated impairments in comparing outcome options in concert with disturbed neural underpinnings of deficient reward processing reflected in disturbed activation in a network comprising medial prefrontal regions such as VMPFC and medial OFC and ventral striatum (Völlm et al., 2007; Peters and Büchel, 2011; Sonuga-Barke and Fairchild, 2012). However, neuroimaging studies directly testing these hypotheses are needed.

## Limitations and Implications for Further Research

This review provides an overview of component-specific frontal dysfunctions underlying impulse control deficits in BPD and ADHD. The implications of the current review are, however, limited by three factors. First, not all studies reviewed clearly stated whether contrasts of interest comprised successful and unsuccessful inhibition trials or whether unsuccessful trials were modeled separately. In addition, in studies with a blocked design error trials are not excluded from the analysis and thus, comparing inhibition vs. non-inhibition blocks entail successful and unsuccessful inhibition trials. This is of crucial importance for at least three reasons. First, patient and healthy control groups might differ with respect to error processing. Second, both successful and unsuccessful inhibition have been associated with activation in overlapping but differential networks comprising VLPFC/insula and ACC (Aron and Poldrack, 2006; Boehler et al., 2010; Erika-Florence et al., 2014). However, as these regions are activated by successful and unsuccessful inhibition to a varying extend and as patients might differ from control groups not only in inhibitory but also in error processing, conflating successful and unsuccessful inhibition trials will most likely bias the results. Third, inhibitory processing is present in unsuccessful inhibition trials, albeit in a less pronounced or weakened form (Boehler et al., 2010). Taken together, not dissociating successful and unsuccessful inhibition trials might distort group differences in brain activation patterns as a function of inhibitory processing. This assumption is underlined by findings from one study assessing behavioral inhibition in ADHD (Sebastian et al., 2012). In that study, patients with ADHD displayed hypofunction of the basal ganglia when contrasting successful stop vs. go trials. However, when contrasting successful vs. unsuccessful stop trials, hypoactivation in a fronto-striatal network comprising VLPFC and insula was observed. Therefore, future neuroimaging studies should clearly distinguish successful and unsuccessful inhibition trials and model brain activity separately for these conditions.

The second limitation concerns the conflation of emotional dysregulation and impulsivity that is present in most neuroimaging studies in BPD. As both emotional dysregulation and impulsivity are clinical hallmarks of BPD, it is comprehensible that in most neuroimaging studies impulsivity was assessed in a context of negative emotions. However, it becomes more and more evident that individuals with BPD are usually not impaired in behavioral inhibition in a neutral setting and this could also apply for some of the other components of impulse control [for a review see Sebastian et al. (2013a)] despite meta-analytic evidence for cognitive/executive deficits categorized in terms of more global cognitive functions like attention or processing speed (Ruocco, 2005). It is therefore crucial to assess the cognitive process of impulse control and its neural underpinnings in a component-specific manner in a neutral setting to finally gain a better understanding of the specificity of impulse control disturbances and their interactions with emotional dysregulation in BPD.

Finally, only an insufficient number of studies are hitherto available to assess frontal dysfunctions along different impulse control components in ADHD and BPD. Especially proactive inhibition and information sampling has barely been studied in both patient groups. In addition, no imaging studies on delay discounting in BPD are to date available. Therefore, implications from this review must be considered as preliminarily. Yet, we are convinced that these interim conclusions provide a basis to understand differences and commonalities of prefrontal cortex dysfunction in impulsive disorders and might serve as a hypothesis for future studies on the systematic assessment of impulse control components in psychiatric conditions.

Future studies should further focus on proactive and reactive inhibitory control as this has not yet been systematically studied in BPD and ADHD, although it is intriguing that highly impulsive subjects act rather reactively impulsive, i.e., by utilizing fewer cues to control their behavior. Only one study used a mixed design to study stimulus interference in ADHD (Banich et al., 2009). This study revealed DLPFC dysfunction to be related to proactive stimulus interference and medial prefrontal dysfunction to be linked to reactive stimulus interference. Only indirect evidence from different studies assessing stimulus interference with either blocked or event-related design in BPD is available (Wingenfeld et al., 2009; Holtmann et al., 2013) The results suggest an opposite pattern in BPD with DLPFC dysfunction underlying reactive stimulus interference and medial prefrontal dysfunction linked to proactive stimulus interference. These preliminary conclusions are, however, speculative and need to be tested in studies using mixed designs allowing for direct comparisons of proactive and reactive impulse control, not only during stimulus interference, but also during other components of impulse control. Future studies should also focus on stress-relatedness of components of impulse controls, given the strong dependence of behavioral alterations on emotional status in this patient group (e.g., Krause-Utz et al., 2013; Cackowski et al., 2014).

## Conclusion

Taken together, patients with BPD exhibit prefrontal dysfunctions across impulse components rather in orbitofrontal and dorsolateral PFC regions, whereas patients with ADHD display disturbed activity mainly in VLPFC and ACC. Prefrontal dysfunctions, however, vary depending on the impulse control component and from disorder to disorder. Although only few but rather insufficient number of studies are hitherto available to reliably assess frontal dysfunctions along different impulse control components in ADHD and BPD, we suggest that such a systematic approach will help to understand prefrontal dysfunctions associated with impulsivity in different psychiatric disorders. Component-specific assessment of impulse control in healthy participants has revealed differential accentuation in activation patterns of the neural impulse control network (Nee et al., 2007; Swick et al., 2011; Sebastian et al., 2013b). Investigation of psychiatric patient groups, however, is still in its infancy. Yet, what we can learn from these studies at this early stage is that deficient impulse control in psychiatric patient groups is multifaceted and so are the neural dysfunctions underlying these disturbances. The identification of cognitive phenotypes along or across diagnostic borders will, however, enable the development of innovative treatment options (e.g., stimulation or feedback based methods) for so far often times’ intractable impulse control deficits.

## Conflict of Interest Statement

The authors declare that the research was conducted in the absence of any commercial or financial relationships that could be construed as a potential conflict of interest.
